# Improving Hand Hygiene Practices to Reduce CLABSI Rates: Nurses Education Integral for Success

**DOI:** 10.5005/jp-journals-10071-23200

**Published:** 2019-07

**Authors:** Sheila Nainan Myatra

**Affiliations:** Department of Anesthesiology, Critical Care and Pain, Tata Memorial Hospital, Homi Bhabha National Institute, Mumbai, Maharashtra, India

## Abstract

**How to cite this article:** Myatra SN. Improving Hand Hygiene Practices to Reduce CLABSI Rates: Nurses Education Integral for Success. Indian J Crit Care Med 2019;23(7):291–293.

Health-care-associated infections are a huge concern in the intensive care units (ICUs) across the globe.^1^ Nearly 90% of catheter-related bloodstream infections are caused by central venous catheters (CVCs).^2^ CVCs are commonly inserted in critically ill patients and play an important role for hemodynamic monitoring and delivery of crucial treatments. Central line-associated bloodstream infection (CLABSI) is a laboratory confirmed bloodstream infection occurring in a patient with a CVC in place for >48 hours that is not related to an infection at any other site.^3^ The 2016 International Nosocomial Infection Control Consortium (INICC) report shows a CLABSI rate of 4.1 per 1,000 central line-days, in 861,284 medical and surgical patients in ICUs in the developing countries, which is almost five times higher than 0.8 per 1,000 central line-days in comparable ICUs from the USA.^4^ CLABSI results in significant morbidity, mortality, increased hospital stay and cost, making prevention crucial for patient safety.^5^

Preventing nosocomial infections pose a great challenge in ICU, because of the multi-drug resistant organisms treated with broad-spectrum antibiotics. Clinical practice guidelines from various organizations are available for prevention of vascular catheter related infections.^3,6^ The Institute for Healthcare Improvement has introduced a “central line bundle” that includes the five best evidence based practices to prevent CLABSI.^7^ This bundle includes hand hygiene, maximal barrier precautions during catheter insertion, skin antisepsis with chlorhexidine, optimal selection of the catheter site, with avoidance of the femoral vein and a daily assessment of line necessity, including the removal of an unnecessary line. Of these components, “hand hygiene” (HH) is the most convenient and cost effective to follow. HH prevents the transmission of pathogens and is a universal strategy to prevent all nosocomial infections. The World Health Organization has proposed a multimodal strategy; which includes five elements called the “Five Moments for Hand Hygiene”, as an evidence based approach to prevent hospital-acquired infections. The five moments include, before touching the patient, before performing any a clean/aseptic procedures, after exposure/risk of body fluid, after touching the patient and after touching the patient surroundings.^8^

There is a risk of acquiring a blood stream infection both during the insertion and maintenance care of CVCs. A systematic review and meta-analysis, which included 79 studies looking at the effectiveness of both of insertion and maintenance bundles for preventing CLABSI in ICU patients over 15 years, found that the implementation of the central-line bundles reduced the incidence of CLABSI.^9^ Implementation of the central line bundle was equally effective in low, middle and high income countries. However, the risk reduction was more when the baseline CLABSI rates were five or higher per 1000 catheter-days as compared to when the CLASBSI rates were lower than this. In addition, the effects of the central-line bundles implementation were sustained over time.^9^ The INICC multidimensional approach for prevention of CLABSI in 35, 650 adult patients hospitalized in 16 Indian ICUs showed a reduction in baseline CLABSI rate of 6.4 CLABSIs per 1000 CL-days, to 3.9 CLABSIs per 1000 CL-days in the next year and this was maintained up to the 36-month follow-up period.^10^ A systematic review of 16 clinical trials in Europe and USA, adopting a multimodal approach to strategies to improve HH, resulted in significant improvement in HH compliance.^11^ Implementation of HH resulted reduced CLABSI rates in Columbian ICUs and a cessation in an outbreak of Acinetobacter.^12^ An Indian study which implemented the INICC multi-dimensional HH in three ICUs showed that the overall adherence to HH increased from 36.9% to 82% over seven years. It was interesting to note that, the HH compliance of doctors was higher than the nurses in India.^13^

Use of evidence based guidelines for the insertion and maintenance of CVCs alone cannot prevent CLABSI. Prevention of CLABSI requires a multi-dimensional approach, which includes behavior modifications of health care professionals through education, assessment of performance, regular feedback, teamwork and improvements in the overall culture of safety. A systematic review of evidence-based measures to prevent CLABSI found that, care bundles coupled with education and the commitment of both staff and institutions, is a strategy that can contribute to decreased rates of central line-associated bloodstream infections among adult patients hospitalized in ICUs.^14^ Four studies from this systematic review addressed educational strategies as the study's main focus and showed a reduction in CLABSI rates. Two among these also addressed cost-effectiveness of educational strategies and suggested that a variety of educational approaches could be cost effective and decrease the hospital cost. A systematic review, which looked at interventions to improve HH compliance in the ICU included 90% of studies which implemented a bundled intervention, of which the most commonly employed strategy was education (78.9%).^15^ A recent Chinese study was able to demonstrate a hospital-wide reduction in CLABSI rates, through a quality improvement programme and multidisciplinary teamwork, an integral part of which was education.^16^

Among the health care workers, nurses have the most direct and continuous role in handling CVCs, being involved with both insertion assistance and maintenance of central lines. Thus they have a unique opportunity to contribute towards preventing these infections.

However, there is lack of research into level of compliance with CLABSI prevention guidelines by nurses internationally. A recent study showed 70% compliance among nurses with CLABSI guidelines.^17^ Low compliance with hand hygiene among nurses has been reported.^18^ An Italian study assessing the knowledge, attitudes, and practice on CLABSI prevention among oncological nurses found several gaps. This study clearly highlights the importance of implementing educational interventions to address the gaps in knowledge and practice to improve the compliance with the use of evidence-based prevention interventions for CLABSI among nurses.^19^

In this journal issue, Mishra et al report the findings of their before and after study involving 34 nurses in a medical ICU in a tertiary hospital in India.^20^ The study involved educating nurses in interventions designed to reduce the catheter related infection rates. The main focus of the education programme was on hand hygiene practices. The workshop included a 30 minute lecture and a 30 minute bedside demonstration of hand hygiene practices and also included basic practices for prevention of CLABSI. A before-after evaluation was carried out to assess the benefits of the educational intervention. In addition; an objective test was conducted among the nurses before and after the workshop; to assess the knowledge of CVC care practices. There was a significant reduction in the CLABSI rates from 12.5 to 8.6 per 1000 catheter days before and after the education session along with a simultaneous decrease in non-compliance with hand hygiene from 53% to 34%. This study highlights the importance of an educational intervention in reducing the CLABSI rates, along with the non compliance rates in HH among nurses. In the objective assessment test conducted to assess the knowledge of the ICU nurses, there was a significant increase in the test marks immediately after the educational intervention, but no significant change in the test marks after a 3 and 6 months duration. The non compliance with hand hygiene also increased 6 months after the intervention. Though there was a 31% reduction in CLABSI rates post intervention, the rates are still high. These findings emphasize the need for ongoing education programmes among nurses.

This study has several limitations, the various elements of the CLABSI bundle were not assessed before and after the intervention. In addition, the severity of illness, morbidity, length of ICU stay, costs, and mortality were not compared in the two periods. Though the emphasis of the educational programme was on HH, general measures for prevention of CLABSI were also covered, hence one cannot say with certainty that the reduction in CLABSI rates was only because of the effect of improved compliance with HH, as it may well be a combination of both. Neverthless, it is important to note that there was a reduction in non-compliance with HH between the two period following the educational intervention.

This study is different from previous studies looking at intervention to reduce CLABSI rates. Most previous studies involved all heath care providers, and were largely based on a bundled approach to reduce CLABSI. This study is unique because it looks at the impact of education only among nurses, showing a reduction in CLABSI rates along with an improvement in HH practices post intervention. In addition, the impact of the educational intervention was also assessed by conducting a pre and post intervention test which showed an improvement in knowledge of CLABSI prevention measures following the intervention. This included an assesment after 6 months on the compliance with HH and the knowledge among nurses, thus assessing if there was a sustained impact of this intervention. Nurses should be at the frontline of any CLABSI prevention programme. This important study highlights the fact that nurses education with the CLABSI prevention bundles, especially hand hygeiee is integral for reducing CLABSI rates and that nursing education should remain ongoing.
